# The impact of vitamin D_3_ intake on inflammatory markers in multiple sclerosis patients and their first-degree relatives

**DOI:** 10.1371/journal.pone.0231145

**Published:** 2020-04-06

**Authors:** Reza Hashemi, Seyed Saeed Hosseini-Asl, Seyed Rafie Arefhosseini, Mohammad Morshedi

**Affiliations:** 1 School of Nutrition and Food Sciences, Tabriz University of Medical Sciences, Tabriz, Iran; 2 Nutrition Research Center, School of Nutrition, Tabriz University of Medical Sciences, Tabriz, Iran; 3 Department of Genetics, School of Medicine, Ardabil University of Medical Sciences, Ardabil, Iran; 4 Department of Biochemistry and Diet Therapy, School of Nutrition and Food Sciences, Tabriz University of Medical Sciences, Tabriz, Iran; Stony Brook University, UNITED STATES

## Abstract

**Background & aims:**

In our previous study, a Seesaw model was proposed for the fluctuation of crucial anti- (IL-10) and pro-inflammatory (Il-6 & IL-17A) cytokines through vitamin D_3_. In this paper, however, it is intended to extend the mentioned model by assessing the expression mRNA levels of IL-27 and TGF-β1 as well as the changes of plasma levels of IL-27, TGF-β1, IL-17A, IL-10, and IL-6 after treatment by vitamin D_3_.

**Method:**

Venous blood samples were drawn from Healthy Participants (HP, n = 25) and First-Degree Relative Participants (FDRP, n = 25) as control groups and Multiple Sclerosis Participants (MSP, n = 25) before and after eight weeks of supplementation with 50000 IU vitamin D_3_. The mRNA expression and plasma concentrations were gauged by using Real-Time PCR and ELISA assay, respectively.

**Results:**

The mRNA surfaces of IL-27, as well as TGF-β1, were up-regulated. However, the plasma levels of TGF-β1, IL-17A, and IL-6 were significantly different among the three groups. In addition, the plasma levels of IL-27, TGF-β1, IL-10, IL-17A, and IL-6 significantly changed following the administration of vitamin D_3_.

**Conclusion:**

The findings of this paper illustrate that anti-inflammatory cytokines could have a key role in immunomodulatory functions due to their anti-inflammatory functions. To conclude, this might contribute to preventing the pathophysiological process of MS. Also, the proposed model could be used as a preventive way on disposed people to multiple sclerosis, particularly in first degree relatives of these patients.

## 1. Introduction

Multiple Sclerosis (MS) is known to be an autoimmune and inflammatory disorder of the Central Nervous System (CNS) which, is recognized by chronic inflammation, demyelination of axons, neuronal injuries, and disabilities affecting approximately 2.5 million people worldwide, the majority of which being women [1]. It is proposed that the combination of genetic and environmental factors are the most significant MS risk factors. Furthermore, a lack of vitamin D_3_ is a determinant risk factor, as well as frequently reported environmental factors in the etiology of MS regardless of the geographical latitude and viral infections [2, 3]. The underlying mechanisms of pathology and etiology of MS have not been dissolved yet, but evidence has presumed that immunomodulatory and anti-inflammatory treatments can delay MS development. According to the prior note, it has been suggested that vitamin D_3_ can have a major role in immune regulation in MS [2, 4].

It has been suggested that perhaps this disorder depends on the dysregulation of pro- and anti-inflammatory cytokines. Pro-inflammatory cytokines can boost the permeability of the Blood-Brain Barrier (BBB), allowing for the neurodegeneration and demyelination of the CNS; while anti-inflammatory cytokines can repress the secretion of pro-inflammatory cytokines [5]. For this reason, it appears that inflammation can play a considerable role in the degeneration of brain axons and neurons in MS patients. Furthermore, evidence has indicated that the ratio between pro- and anti-inflammatory is high in MS patients with a vitamin D_3_ deficiency. Vitamin D_3_ has a probable immune-modulatory effect which, changes the equilibrium between anti- and pro-inflammatory interleukins in favor of anti-inflammatory cytokines. So, the lack of vitamin D_3_ is possible to cause inflammatory situations in MS patients [2, 4, 6].

Interestingly, Interleukin-27 (IL-27) possesses a dual function or pleiotropic, as anti- or pro-inflammatory properties, in a variety of autoimmune diseases such as MS. Primarily, IL-27 was proposed to provoke pro-inflammatory state by meliorating T-helper 1 (Th1) differentiation from naïve T cells in early immune response via Signal Transducer and Activator of Transcription 1 (STAT1) [7–9]; however, subsequent work using human and animal models of MS have indicated that IL-27 has considerable inhibitory influences on Th1, Th2, and Th17 (IL-17A and IL-17F) subsets of T cells, and on Antigen-Presenting Cells (APCs) function. On the same note, a few studies have indicated the protective role of IL-27 in Th1/Th17-mediated immune diseases [10]. Nevertheless, recent studies have emphasized the anti-inflammatory aspect of IL-27 and achieved further data by promoting inducible regulatory T (T-reg) cells to secrete IL-10. Also, IL-27 powerfully provoked the differentiation of Cluster of Differentiation 4+ (CD4+) and CD8+ effector T cells to IL-10, T-bet, and Interferon-ɣ (IFN-ɣ) [7, 8, 11, 12]. Moreover, Transforming Growth Factor-β (TGF-β) has been recognized as a pleiotropic cytokine with remarkable immunomodulatory impacts which, is mainly produced by T-reg cells to manage autoimmune diseases. However, this paradoxical role of TGF-β relies on the cell surface co-receptors, the differentiation state of T cells, and incitement situation [13, 14]. Equally important, IL-27 works together with TGF-b to further enhance Type 1 regulatory (Tr1)-like cells that produce large amounts of IL-10 and IFN-ɣ and decrease IL-17A and IL-17F production [15]. Nevertheless, it is notable that TGF- β1 promotes differentiation of naïve CD4+ T cells into Foxp3^+^ Tregs through signal STAT5, thereby increasing the expression of anti-inflammatory cytokines like IL-10. Additionally, TGF- β1 plays a preventative role in terms of the entrance of sensitized T cells into the CNS and consequently quells the production of pro-inflammatory cytokines [14, 16, 17]. Naïve CD4+ T cells can nonetheless be converted into immature Th17 in the presence of IL-6 and via signal STAT3. Subsequently, pathogenic maturation of Th17 cells, such as IL-17A, IL-17F, and IL-22 are released in the presence of IL-23 [17]. Up to now, the limited studies have researched the relation between vitamin D_3_ and expression of IL-27 and TGF- β1 in MS patients. Vitamin D_3_ could both directly and indirectly, upregulate the expression of IL-27 and TGF- β1. All the same, they are not enough and need to clarify more the precise role of vitamin D_3_ in regulating them [13, 18, 19].

Due to the highlighting role of genetics in MS which has somewhat remained obscure, in our previous [5] and present studies, attempts were conducted to introduce nutrition genomics or nutrigenomics as a new potential therapeutic method in afflicted patients and prohibiting the illness in other people, especially in the first-degree relatives of MS patients. According to IL-27 and TGF- β1 being key regulators of IL-10, IL-17A, and IL-6, this paper highlights that the up-regulation of IL-27 and TGF- β1 by vitamin D_3_ increases the expression of IL-10, as well as down-regulates IL-17A and IL-6.

## 2. Experimental procedures

### 2.1. Ethical codes and patient consent

The study protocol was reviewed and approved by the Iranian Registry of Clinical Trials (IRCT20100407003655N4) and Ethics Committee of Tabriz University of Medical Sciences, Iran (code of ethics: IR.TBZMED.REC.1397.608) before the recruitment of participants. Written informed consent was also received from all patients and control groups; besides, they were given a choice to withdraw from the study upon their willingness.

### 2.2. Study groups and intervention

Sample selection and supplementation design were discussed in our previous publication [5]. In short, the subjects registered into the present study were divided into three groups: 1) MS participants (MSP, n = 25) as the first treatment (case) group along with two control groups including 2) First degree relatives of MS participants as the second group (FDRP, n = 25) such as their daughter, son, brother, or sister and 3) Healthy participants (HP, n = 25) as the third group. The study was conducted on the 19th of February, 2017, and ended on the 10th of June, 2017. To be included in this study, participants were 1) between 20 to 40 years of age, 2) willing to take part in the study, 3) able to donate blood samples, and 4) of no family kinship with MS patients for HPs. Additionally, the exclusion criteria were: 1) taking medicines that interact with vitamin D_3_, 2) Malabsorption, 3) gestation and lactation, and 4) vitamin D_3_ and calcium supplementation in the last one month. After applying the inclusion and exclusion criteria, a total number of 25 participants were then randomized into each group by using a simple random sampling method.

The demographic and disease characteristics of the study have recapped in Table 1. The first and second groups were also picked out from Ardabil MS Society and, the patients were confirmed based on McDonald’s criteria and by a certified neurologist [17]. What is more, the HP was selected from the Ardabil University of Medical Sciences. All groups pulled down 50,000 IU of vitamin D_3_ orally every Friday with their lunch breaks for two months. As previously described, before and after the administration of vitamin D_3_, five milliliters whole blood samples were drawn from the selected participants, and the serum levels of 25-(OH) vitamin D_3_ were measured immediately after serum separation by using electrochemiluminescence (ECL) assay [5]. A certified laboratory performed all measurements in the Genetics Laboratory at the Imam Khomeini Hospital and Biochemistry laboratory in the Research Center, Ardabil University of Medical Sciences.

**Table 1:** Demographic and disease characteristics.

Variables	MSP (n = 25)	FDRP (n = 25)	HP (n = 25)
MS family*			
Brother	1(4%)	5(20%)	-
Sister	3(12%)	12(48%)	-
Daughter	1(4%)	5(20%)	-
Son	-	3(12%)	-
MS History*			
Yes	5(20%)	25(100%)	-
No	20(80%)	-	25(100%)
Duration of disease(year)**	8.1±5.8	-	-
Sex*			
Female	21(84%)	17(68%)	20(80%)
Male	4(16%)	8(32%)	5(20%)
Age(year)**	32.6±6	27.4±6	31.7±4.3

The data are presented as frequency (percent) for categorical * variables and as mean ± SD for numeric normal ** variables. Abbreviation: MSP, Multiple Sclerosis Participants; FDRP, First-Degree Relative Participants; HP, Healthy Participants.

### 2.3. RNA isolation and real-time PCR analysis for IL-27 and TGF-β1

The MN kit (MACHEREY-NAGEL, Germany) was applied to extract total RNA of Peripheral Blood Mononuclear Cells (PBMCs), which were collected in anti-coagulant EDTA tubes, based on the manufacturer’s orientations. Purity, concentration, and integrity of total RNA were confirmed by nano-drops (Thermo Scientific), gel electrophoresis, and spectrometry. Afterward, the synthesis of cDNA was made with five μg of the total RNA by using a Random Hexamer Primer through HyperScript^™^ Reverse Transcriptase (GeneAll, South Korea) in 20 μl total reaction mixture. The mRNA expression levels of IL-27 and TGF-β1 genes were assessed with appropriate primers and probes and were normalized with the housekeeping gene β-actin. Suitable primers and probes sequences for IL-27p28 (Forward: GGAGCGTCTCTGCTTCATCT; Reverse: AGCTGCATCCTCTCCATGTT; Probes: FAM-CGCTTCAGCCCTTCCATGCC) [20], TGF-β1: (Forward: CAGCAACAATTCCTGGCGATA; Reverse: AAGGCGAAAGCCCTCAATTT; Probe: FAM- CTGCTGGCACCCAGCGACTCG, and β-actin (Forward: TCACCCACACTGTGCCCATCTACGA; Reverse: CAGCGGAACCGCTCATTGCCAATGG; Probe: FAM-ATGCCCTCCCCCATGCCATC) [21]—as a housekeeping control- were selected. Quantitative Real-time PCR was fulfilled for mRNA expression on a Roche Light cycler 96 (version: 1.1.0.1320, Germany) using 12.5 μl of RealQ Plus 2x Master Mix for probe (Amplicon, Denmark), 250 nM of the TaqMan probe, 500 nM of each forward and reverse primers, and 4 μl of synthesized cDNA solution in total volume of 25 μl. Finally, the amplification of PCR was concluded through the following schedule: an initial warming step for ten minutes at 94°C, followed by a denaturation step at 94°C for fifteen seconds, and finally, an annealing/extension one for sixty seconds at 60°C.

### 2.4. Measurement plasma levels of IL-17A, IL-6, IL-10, IL-27, and TGF- β1 by ELISA

Here, five milliliters of the entire blood samples were taken at the beginning and the end of the intervention period and were collected in anticoagulant tubes containing ethylenediaminetetraacetic acid (EDTA), the blood samples were centrifuged at room temperature for ten minutes at 3000 rpm to separate the plasma, following which the plasma samples were immediately frozen at– 80°C until analyzed. According to the manufacturer's instructions, the plasma concentration of IL-17A, IL-6, IL-10, IL-27, and TGF- β1 from MSP, FDRP, and HP were determined by using Enzyme-linked Immunosorbent Assay (ELISA) kits (Diaclone, SAS, France). The kits’ sensitivities for these cytokines were 2.3 pg/ml, 2 pg/ml, 5 pg/ml, 12.8 pg/ml, and 9 pg/ml, respectively.

### 2.5. Statistical analysis

The statistical analysis was fulfilled through SPSS Statistics Version 23.0 Software (SPSS Inc., Chicago, IL, USA) and GraphPad Prism 8 (GraphPad Software, Inc., San Diego, CA, USA). Comparisons among groups were assessed by using a One-way analysis of variance (ANOVA), followed by post-hoc Tukey’s test. After that, within-group comparisons were surveyed by the Paired t-test. The data were then expressed in a mean±standard deviation (SD) format for the normal numeric variables. Notably, the fold change expression of each gene was calculated by a ratio = 2−ΔΔCT formula, and the amplification efficiency between the target mRNA expression and β-actin was measured by the ΔΔCT method [22]. Finally, P-values of less than 0.05 was considered significant in all of the analyses.

## 3. Results

### 3.1. Clinical features

**Fig 1:**
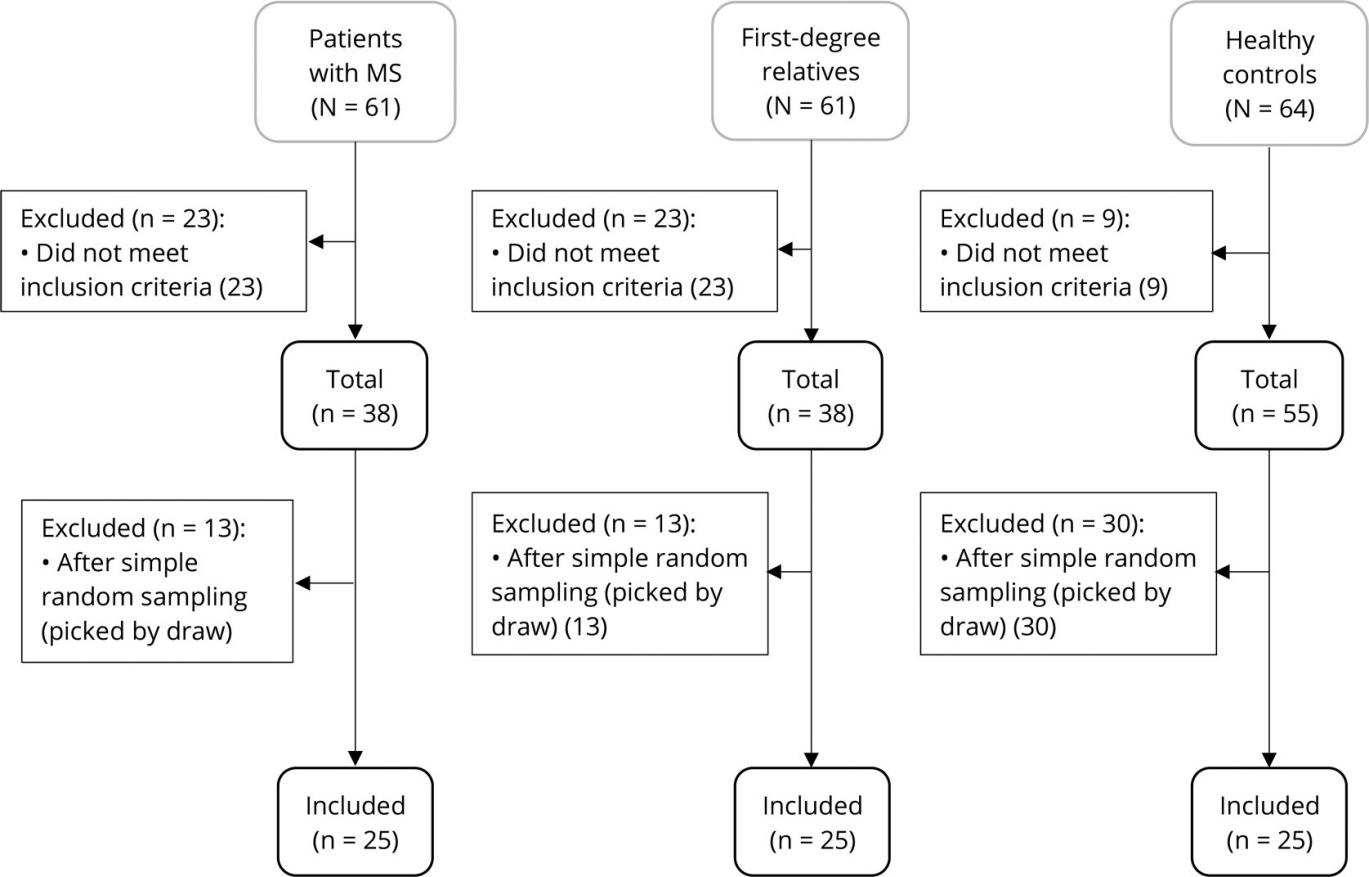
Enrollment and selection of participants allocated to groups by simple random sampling.

The flow diagram has been illustrated in Fig 1 as well as vitamin D_3_ serum levels and the expression mRNA levels of IL-17A, IL-6, and IL-10 in S1 Table [5].

### 3.2. The mRNA expressions of IL-27 and TGF- β1 before and after intervention

**Fig 2:**
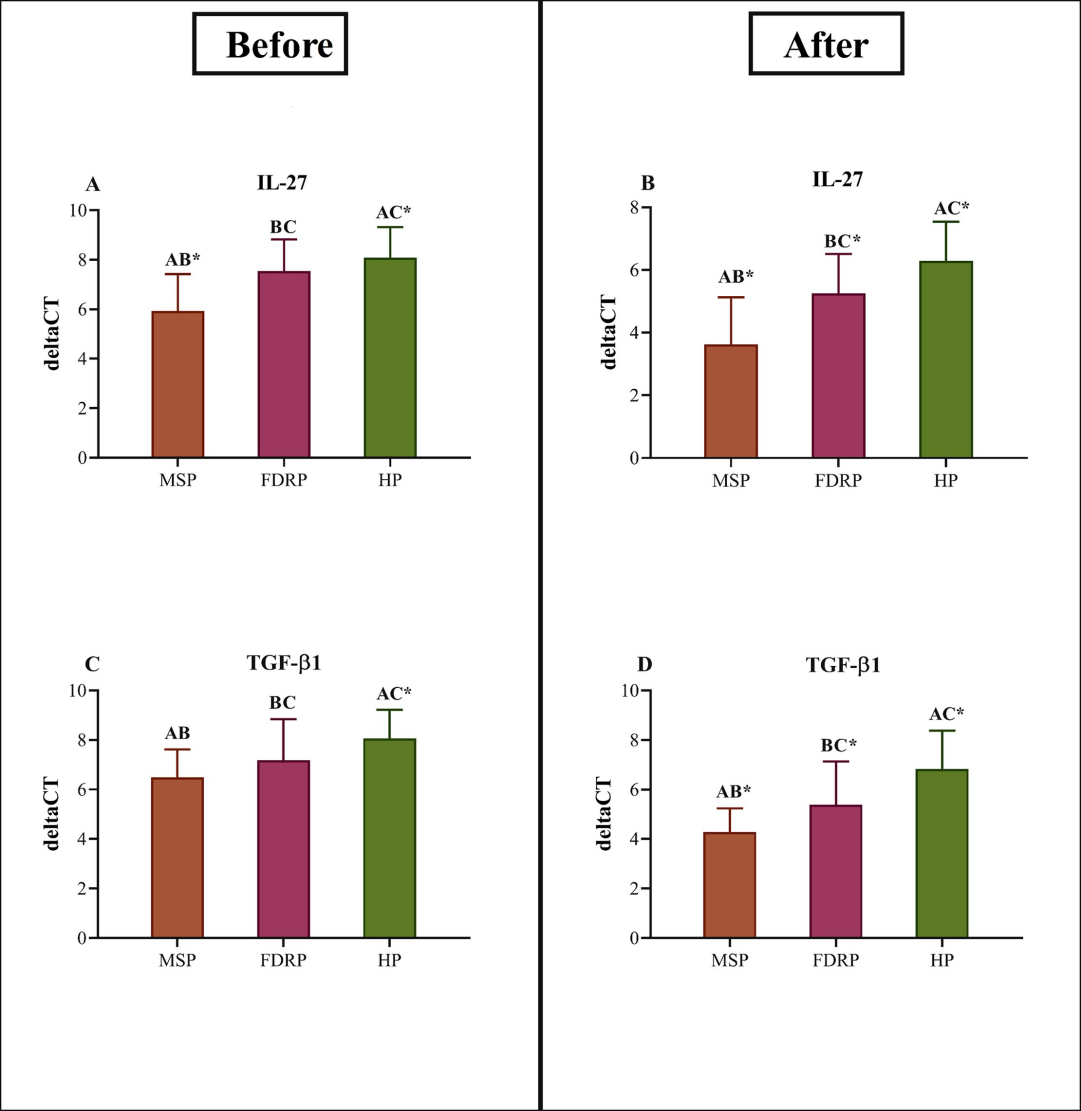
The outcome of IL-27 & TGF-β1 mRNA expression analysis. Impacts of vitamin D_3_ administration on mRNA expression of anti-inflammatory cytokines (n = 25 per group). (A-D) IL-27 and TGF-β1 mRNA expression surfaces of Multiple Sclerosis Participants (MSP), First Degree Relatives Participants (FDRP), and Healthy Participants (HP). In comparison, among groups, MSP, FDRP, and HP have been identified by A, B, and C, respectively. One-way ANOVA and then, post-hoc Tukey’s test, was applied. The data were then represented as mean ± standard deviation (SD), and * P<0.05 was considered as statistically significant between groups.

Before the intervention, the test of one-way ANOVA elucidated differences in the PBMCs levels of IL-27 (*P*<0.001) and TGF-β1 (*P*<0.001) among the study groups. Since there is an inverse relationship between ΔCT and mRNA expression, the results of this study clarified the mRNA expression of either IL-27 or TGF-β1 are high in MSP, FDRP, and HP, respectively [ΔCT IL-27: MSP: 5.93±1.49 vs. FDRP: 7.54±1.28 vs. HP: 8.09±1.23; ΔCT TGF-β1: MSP: 6.49±1.13 vs. FDRP: 7.18±1.66 vs. HP: 8.06±1.16]. Then, the post-hoc Tukey’s test also revealed dramatic differences between MSP & FDRP (*P*<0.001) and MSP & HP (*P*<0.001); but no statistical differences between FDRP & HP (*P* = 0.320) in IL-27. In TGF-β1, the data indicated compelling differences between MSP & HP (*P*<0.001). On the plus side, differences were seen between MSP & FDRP (*P* = 0.171) and FDRP & HP (*P* = 0.060) in TGF-β1, yet none was statistically significant (Table 2 & Fig 2A and 2C).

**Table 2:** Interleukin ΔCT in before and after eight weeks of treatment with vitamin D_3_.

Variables	MSP, n = 25		FDRP, n = 25		HP, n = 25		P*	
	Before	After	Before	After	Before	After	Before	After
**IL-27 (mean±SD)**	5.93±1.49	3.63±1.50	7.54±1.28	5.25±1.26	8.09±1.23	6.29±1.25	<0.001	<0.001
P**	AB<0.001	AB<0.001	BC = 0.320	BC = 0.023	AC<0.001	AC<0.001		
Fold changes†	-	5.4	-	4.7	-	3.5		
P***	-	<0.001	-	<0.001	-	<0.001		
**TGF-β1(mean±SD)**	6.49±1.13	4.28±0.96	7.18±1.66	5.39±1.74	8.06±1.16	6.83±1.55	<0.001	<0.001
P**	AB = 0.171	AB = 0.023	BC = 0.060	BC = 0.002	AC<0.001	AC<0.001		
Fold changes†	-	4.3	-	3.5	-	2.7		
P***	-	<0.001	-	0.002	-	0.007		

The data are represented as mean ± standard deviation (SD). One-way ANOVA and then, post-hoc Tukey’s test were applied to the analysis of differences between groups. Within-group comparisons of mRNA expression surfaces of IL-27 and TGF-β1 were also fulfilled by using the paired t-test. Between-group differences, pairwise comparisons (Tukey’s test), and within-group differences were shown by P*, P**, and P***, respectively. Likewise, MSP, FDRP, and HP were represented by A, B, and C. P<0.05 shows the statistical significance, and the sign † stands for the expression surfaces of cytokines in comparison with the baseline values, according to the ratio formula (2-ΔΔCT). Abbreviations: ANOVA, analysis of variance; IL, Interleukin; TGF-β1: transforming growth factor-β; MSP, Multiple Sclerosis Participants; FDRP, First-Degree Relative Participants; HP, Healthy Participants.

Furthermore, the eight-week treatment with vitamin D_3_ indicated statistical differences among study groups in terms of mRNA expression surfaces of IL-27 (*P*<0.001) and TGF-β1 (*P*<0.001) in PBMCs. Herein, the mRNA expression of IL-27 and TGF-β1 have up-regulated in all groups but MSP holds the highest mRNA expression in relative FDRP and HP [ΔCT IL-27: MSP: 3.63±1.50 vs. FDRP: 5.25±1.26 vs. HP: 6.29±1.25; ΔCT TGF-β1: MSP: 4.28±0.96 vs. FDRP: 5.39±1.74 vs. HP: 6.83±1.55]. What is more, pair-wise comparisons explicated differences between each two groups in IL-27 (MSP & FDRP: *P*<0.001, FDRP & HP: *P* = 0.023, MSP & HP: *P*<0.001) and TGF-β1 (MSP & FDRP: *P* = 0.023, FDRP & HP: *P* = 0.002, MSP & HP: *P*<0.001 (Table 2 & Fig 2B and 2D).

Additionally, the outcomes of paired t-test analyses–in all study groups, especially in MSP- indicated that the administration of vitamin D_3_ up-regulates the production of IL-27 and TGF-β1, as anti-inflammatory cytokines. The observed fold changes in MSP were 5.4 for IL-27 (*P*<0.001) and 4.3 for TGF-β1 (*P*<0.001), and the obtained fold changes for FDRP were also reported 4.7 for IL-27 (*P*<0.001) and 3.5 for TGF-β1 (*P* = 0.002). After all, the fold changes in HP were 3.5 for IL-27 (*P*<0.001) and 2.7 for TGF-β1 (*P* = 0.007) (Table 2).

### 3.3. The plasma levels of inflammatory parameters before and after supplementation

**Fig 3:**
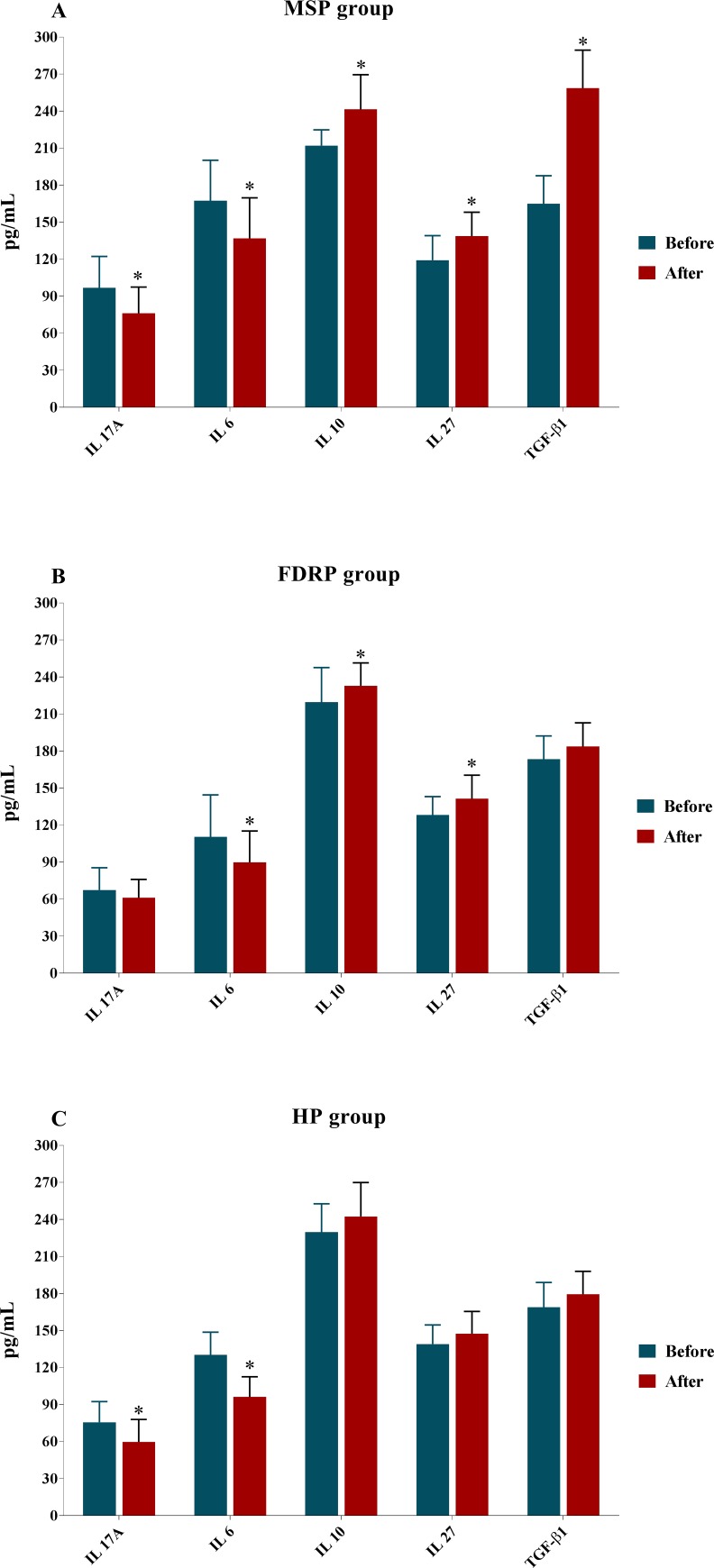
The outcome of IL-27, TGF-β1, IL-17A, IL-10 & IL-6 protein levels analysis. Effects of vitamin D_3_ administration on plasma concentrations of cytokines in Multiple Sclerosis Participants (MSP), First Degree Relatives Participants (FDRP), and Healthy Participants (HP) (n = 25 per group). One-way ANOVA and then, post-hoc Tukey’s test, was applied. We observed that supplementation with vitamin D_3_ had significantly effect in changing plasma levels of IL-27, TGF-β1, IL-17A, IL-10 & IL-6 in MSP group (A), while, only the plasma levels of IL-6, IL-10 & IL-27 in FDRP group (B) and IL-17A & IL-6 in HP group (C) changed. Mean ± standard deviation (SD) and asterisk (*) represents the differences before and after supplementation, also, P<0.05 was considered as statistically significant between groups.

Before beginning the supplementation of vitamin D_3_, the plasma levels of IL-27 and IL-10 in MSP were considerably lower than that FDRP and HP [(IL-27: MSP: 119.19±19.90 vs. FDRP: 128.16±14.95 and HP: 139.01±15.59; *P*<0.001; Table 3 & Fig 3) (IL-10: MSP: 212.10±12.74 vs. FDRP: 219.64±27.97 and HP: 229.77±22.96; *P* = 0.022; Table 3 & Fig 3)] while, no statistical difference was found in plasma concentrations of TGF-β1 among three groups (MSP: 164.92±22.75 vs. FDRP: 173.45±18.85 and HP: 168.94±20; *P* = 0.347; Table 3 & Fig 3). Interestingly, higher IL-6 and IL-17A plasma levels were found in MSP in comparison with FDRP and HP [(IL-6: MSP: 167.37±32.82 vs. FDRP: 110.39±34.04 and HP: 130.27±18.42; *P*<0.001) (IL-17A: MSP: 96.76±25.57 vs. FDRP: 67.44±18 and HP: 75.72±16.64; *P*<0.001) Table 3 & Fig 3]. Also, as is represented by the post-hoc Tukey’s test in Table 3, there were significant differences between MSP & HP (*P*<0.001) in IL-27, between MSP & FDRP (*P*<0.001) and MSP & HP (*P* = 0.001) in IL-17A; MSP & HP (*P* = 0.017) in IL-10; MSP & FDRP (*P*<0.001), MSP & HP (*P*<0.001), and between FDRP and HP (*P* = 0.049) in IL-6.

**Table 3:** Plasma levels (pg/ml) of cytokines before and after eight weeks of treatment with vitamin D_3_.

Variables	MSP, n = 25		FDRP, n = 25		HP, n = 25		P*	
	Before	After	Before	After	Before	After	Before	After
**IL-27** (mean±SD)	119.19±19.90	138.81±19.39	128.16±14.95	141.35±19.05	139.01±15.59	147.35±18.13	<0.001	0.266
P**	AB = 0.155	AB = 0.883	BC = 0.068	BC = 0.502	AC<0.001	AC = 0.252		
P***	-	0.001	-	0.002	-	0.096		
**TGF-β1**(mean±SD)	164.92±22.75	258.58±30.83	173.45±18.85	183.74±19.09	168.94±20	179.41±18.55	0.347	<0.001
P**	AB = 0.314	AB<0.001	BC = 0.720	BC = 0.793	AC = 0.770	AC<0.001		
P***	-	<0.001	-	0.109	-	0.069		
**IL-10** (mean±SD)	212.10±12.74	241.45±28.06	219.64±27.97	232.88±18.48	229.77±22.96	242.39±27.51	0.022	0.341
P**	AB = 0.455	AB = 0.453	BC = 0.245	BC = 0.378	AC = 0.017	AC = 0.990		
P***	-	<0.001	-	0.026	-	0.066		
**IL-17A** (mean±SD)	96.76±25.57	76.10±21.39	67.44±18	61.10±14.96	75.72±16.64	59.71±18.28	<0.001	0.004
P**	AB<0.001	AB = 0.014	BC = 0.330	BC = 0.962	AC = 0.001	AC = 0.007		
P***	-	0.024	-	0.204	-	0.001		
**IL-6** (mean±SD)	167.37±32.82	136.83±32.92	110.39±34.04	89.87±25.39	130.27±18.42	96.21±16.34	<0.001	<0.001
P**	AB<0.001	AB<0.001	BC = 0.049	BC = 0.662	AC<0.001	AC<0.001		
P***	-	<0.001	-	0.003	-	<0.001		

The data are represented as mean ± standard deviation (SD). One-way ANOVA and then, post-hoc Tukey’s test were applied to the analysis of differences between groups. Within-group comparisons of mRNA expression surfaces of IL-27 and TGF-β1 were also fulfilled by using the paired t-test. Between-group differences, pairwise comparisons (Tukey’s test), and within-group differences were shown by P*, P**, and P***, respectively. Likewise, MSP, FDRP, and HP were represented by A, B, and C. P<0.05 shows the statistical significance, and the sign † stands for the expression surfaces of cytokines in comparison with the baseline values, according to the ratio formula (2-ΔΔCT). Abbreviations: ANOVA, analysis of variance; IL, Interleukin; TGF-β1: transforming growth factor-β; MSP, Multiple Sclerosis Participants; FDRP, First-Degree Relative Participants; HP, Healthy Participants.

The changed plasma levels of IL-27, TGF- β1, IL-10, IL-17A, and IL-6 after the administration of vitamin D_3_ are illustrated in Table 3 and Fig 3. It is worth mentioning that in this period, the plasma levels of TGF- β1 [MSP: 258.58±30.83 vs. FDRP: 183.74±19.09 and HP: 179.41±18.55; (*P*<0.001)], IL-17A [MSP: 76.10±21.39 vs. FDRP: 61.10±14.96 and HP: 59.71±18.28; (*P* = 0.004)], and IL-6 [MSP: 136.83±32.92 vs. FDRP: 89.87±25.39 and HP: 96.21±16.34; (*P*<0.001)] were significantly different among the three groups. Meanwhile, serious differences were not observed across MSP, FDRP, and HP in IL-27 [MSP: 138.81±19.39 vs. FDRP: 141.35±19.05 and HP: 147.35±18.13; (*P* = 0.266)] and IL-10 [MSP: 241.45±28.06 vs. FDRP: 232.88±18.48 and HP: 242.39±27.51; (*P* = 0.341)]. Nevertheless, pair-wise comparisons indicated differences between MSP & FDRP (*P*<0.001) and MSP & HP (*P*<0.001) in TGF- β1; MSP & FDRP (*P* = 0.014) and MSP & HP (*P* = 0.007) in IL-17A; MSP & FDRP (*P*<0.001) and MSP & HP (*P*<0.001) in IL-6.

On top of this, the paired T-test elucidated that IL-27 (*P* = 0.001), TGF- β1 (*P*<0.001), and IL-10 (*P*<0.001) up-regulate, and in contrast, IL-17A (*P* = 0.024), and IL-6 (*P*<0.001) down-regulate the treatment with vitamin D_3_ in MSP, whereas TGF- β1 (*P* = 0.109) and IL-17A (*P* = 0.204) in FDRP as well as IL-27 (*P* = 0.096), TGF- β1 (*P* = 0.069), and IL-10 (*P* = 0.066) in HP do not represent any statistical indications of any changes in the plasma levels (Table 3).

## 4. Discussion

**Fig 4:**
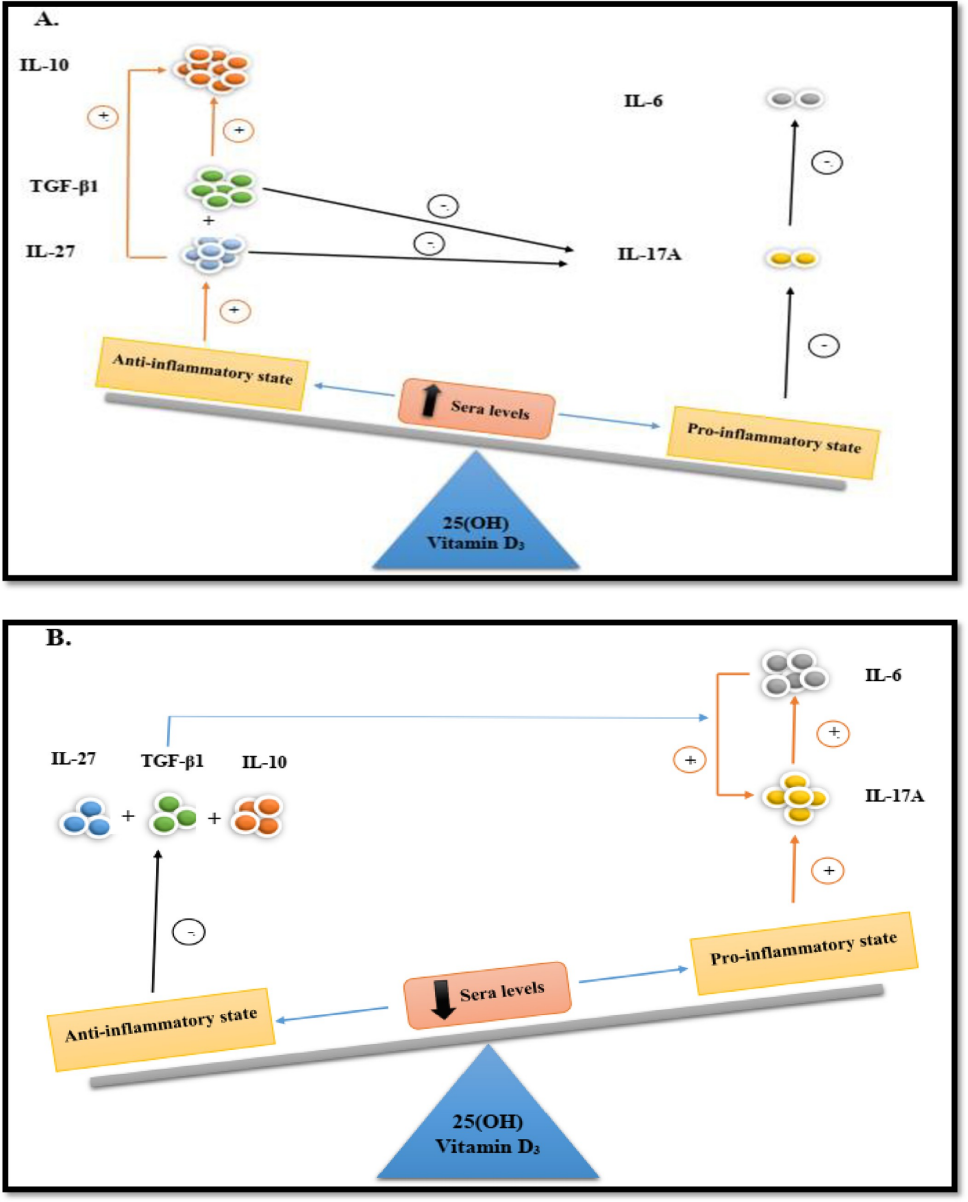
Seesaw model. **(A):** When the sera level of vitamin D is sufficient, the anti-inflammatory state will come true. Thereby, at the first step, the expression levels of IL-27 and TGF-β1 augment. Then, the effectiveness of these cytokines has been represented separately by 1. negative feedback on the IL-17A expression, and 2. Positive feedback on the IL-10 expression as a major anti-inflammatory. After that, the pro-inflammatory state is prohibited. **(B):** Conversely, pro-inflammatory cytokines, especially IL-17A, increases during the deficiency level of vitamin D_3_. IL-17A mediates BBB dysfunction (so, pro-inflammatory cytokines can cross the BBB) and induces secretion of IL-6 by BBB endothelial. However, after the production of IL-6 and in the presence of TGF-β1 in inflammation tissue, IL-6 has positive feedback on increasing IL-17A secretion, too. TGF-β1: Transforming Growth Factor-β1, BBB: Blood-Brain Barrier, IL: Interleukin.

The main purpose of the study in hand was to survey IL-27 and TGF-β1 mRNA expression at the molecular level along with the protein levels of IL-27, TGF-β1, IL-17A, IL-10, and IL-6 concerning the potential impression of vitamin D_3_ in gene expression and immune system. The present paper was designed based on two control groups, including FDRP and HP, alongside MSP to investigate the prominent role of vitamin D_3_ in MS patients. In our previous study [5], schematic primitives, so-called the Seesaw model, were proposed for fluctuation of IL-10 as a major anti-inflammatory as well as IL-17A and IL-6 as a crucial pro-inflammatory cytokines by vitamin D_3_ which clarified that the up-regulation of IL-10 and down-regulation of IL-17A and IL-6 had occurred at adequate sera levels of vitamin D_3_. In this study, the Seesaw model was extended through entrancing IL-27 and TGF-β1 which have been introduced as key factors in the production of IL-10, IL-17A, and IL-6 (Fig 4), and their possible roles in misadjusting pro- and anti-inflammatory state via alteration of vitamin D_3_ sera level were elucidated as well.

This study indicated that the protein concentrations of anti-inflammatory cytokines (IL-27, TGF- β1, and IL-10) in MSP had uplifted with an eight-week treatment with vitamin D_3_ while the plasma levels of pro-inflammatory cytokines (IL-17A and IL-6) had decreased in this group. Interestingly, the cytokines-mediated immunity exerts crucial effects on the pathophysiological progress of autoimmune diseases, such as MS. The aberrant expression of pro- and anti-inflammatory cytokines may be the underlying reason for autoimmune disorders based on the reports [23]. Since MS is an autoimmune disorder characterized by neuronal damage, demyelination, and chronic inflammation, it seems that cytokines are significant inflammation mediators. In particular, IL-27 and TGF- β modify inflammation and can be secreted directly at inflammatory sites in the CNS during MS [16, 24]. In agreement with this paper’s findings, Tang *et al*. [7] explored the role of IL-27 in MS patients and reported that plasma and mRNA expression levels of IL-27 had declined in MS patients. Besides, they indicated that plasma levels of IL-27 are negatively correlated to the percentages of circulating Th17 or plasma IL-17 concentrations in patients, suggesting its involvement in the pathophysiological process of MS.

Moreover, the same results were reported by Babaloo *et al*. [25] in which the expression level of IL-27 had decreased against increasing IL-17A in untreated MS patients compared to the healthy controls. Accordingly, they have suggested a suppressive role of IL-27 on the inflammatory process of MS. In an animal study, Mohammadi-Kordkhayli *et al*. [19] evaluated the impact of vitamin D supplementation on IL-27 expression levels in the experimental autoimmune encephalomyelitis (EAE) and proved that compared to a control group, the expression level of IL-27 P28 and IL-27 EBI3 was powerfully up-surged in vitamin D-treated group. Their study suggests that IL-27 via promoting IL-10 secretion and suppressing the inflammatory effects of IL-17A plays a pivotal role in ameliorating clinical symptoms and the development of MS disorder. It appears that cell differentiation of either T-reg or Th17 depends on TGF-β. Interestingly, naïve T cells become the T-reg cell in the presence of TGF- β (without IL-6), while, in the presence of IL-6, they lead to Th-17 cell differentiation [26]. In other words, the TGF- β/IL-6 ratio may be a remarkable factor in differentiating naïve CD4^+^ cells to Th17 or T-reg cells [27]. Ahangar-Parvin *et al*. [13] illustrated that the expression of TGF-β up-regulates in EAE mice after vitamin D_3_ supplementation. Interestingly enough, a similar finding has been demonstrated by Aivo *et al*. [18] in MS patients. On top of this, Rollnik *et al*. [28] illuminated that the expression level of TGF- β1 had declined in MS patients’ sera compared with the control group and Ishikawa *et al*. [29] confirmed that TGF-β1 was able to improve clinical EAE and inhibit relapse phase in them.

Nevertheless, in several studies, contradictory results have also been reported. For instance, Naderi *et al*. [30] found that the plasma level of IL-27 in MS patients had up-surged in comparison with healthy controls, and Pot *et al*. [31] also indicated these results in cerebrospinal fluid (CSF). Concurrently, Lalive *et al*. [24] had acknowledged an increase in IL-27 levels in the CSF but not in the sera of MS patients compared with healthy subjects. Additionally, Farsani *et al*. [32] investigated the effect of vitamin D_3_ on the expression level of the TGF-β1 gene in PBMCs of MS patients in comparison with healthy control, but they did not observe any remarkable alterations in gene expression before and after treatment. Moreover, in another study conducted by Isik *et al*. [33], the association between TGF- β1 expression and vitamin D_3_ deficiency was assessed in patients who had been referred to Endocrinology and Metabolic Diseases Clinic which resulted in that TGF- β1 was negatively correlated with vitamin D_3_, particularly in patients with lower than 5 ng/ml 25(OH) vitamin D_3_ levels. It is interesting to note that a variety of factors could contribute to such inconsistency outcomes as reported in various studies including sample size and various geographical regions, cell types, genetic inheritance in participants and the crucially double-faced or pleiotropic role of IL-27 and TGF- β1 and, in some of studies, polymorphisms in Vitamin D Receptor (VDR) genes [6, 13, 30, 34].

Based on the accumulated data of our present and previous [34] studies, another major conclusion is related to the key role of IL-27 and TGF- β1 in the expression of pro- and anti-inflammatory cytokines in human immune system, especially in terms of chronic inflammation of the central nervous system like MS (Fig 4). Following this, based on the observed differences and the literature cited, which has clarified a relationship between the cytokines, it suggests that IL-27 and TGF- β1 are key regulators of IL-10, IL-17A, and IL-6 production. Increasing the expression of IL-27 and TGF- β1 leads to a sufficient intake of vitamin D_3,_ and two crucial mechanisms occur in the immune system that creates anti-inflammatory states: 1) both of which directly affect the expression of IL-10, thereby leaving positive feedback on up-regulation of major anti-inflammatory cytokine (IL-10); 2) IL-27 and TGF- β1 have inhibiting impacts on the expression level of IL-17A and result in negative feedback on up-regulation of this pro-inflammatory interleukin. In this regard, Fitzgerald *et al*. [35] elucidated that the up-regulation of IL-10 through increasing the expression of IL-27 was related to less production of IL-17, and exogenous IL-27 could decline the severity of adoptively transferred EAE by a pathway which could link to IL-10. On the other hand, IL-27 could directly influence the regulatory T cell population to produce more IL-10 [8, 15]. Furthermore, more studies reported that IL-27 could quell inflammation state through prohibiting the function and the differentiation of Th17 cells, particularly repression to the secretion of IL-17A via JAK1-STAT1 [36], followed by ameliorated clinical symptoms and development of MS disease [11, 19, 35]. Various studies have illustrated the role of TGF- β1 in inciting the production of IL-10 [32, 37, 38], and also, it has become elucidated that TGF-β1 can trigger IL-10 through Smad4 pathway [16]. Following the same issue, TGF- β1 could repress the differentiation of naive CD4+ T cells into pathogenic Th17 ones and down-regulate the mRNA expression of IL-17 due to the immunosuppressive traits [39–41]. However, there are contradictory results about the impact of TGF-β1 on the differentiation of Th17 to IL-17 in a way that some of them indicated that TGF- β1 was able to trigger the differentiation of Th17 to IL-17 [42, 43]. Moreover, the production of IL-17A had increased in the pro-inflammatory state due to the deficiency level of vitamin D_3_ [44]. After that, IL-17A could mediate BBB dysfunction (so, pro-inflammatory cytokines could cross the BBB) and induce the secretion of IL-6 by BBB endothelial. What is more, the presence of TGF-β1 in inflammation tissue leading to the induction of FOXP3 expression in peripheral T cells together with IL-6 could stimulate T cell differentiation into Th-17 cells, which–as a subsequence—could increase the surface of IL-17A production [45, 46]. Additionally, the composition of IL-6 and TGF- β1 could up-regulate RORγt mRNA, acting as a key regulator of the Th17 cell differentiation and activation of the STAT3 transcription factor [47, 48].

According to these findings, the Seesaw model—expressing the importance of the sera level of vitamin D_3_ in fluctuation and balance of anti- and pro-inflammatory cytokines, as well as this model—could highlight the significance of IL-27 and TGF- β1 in preventing inflammation state in the immune system, particularly in MS patients. Consolidation and appropriate equilibrium of the schematic Seesaw model by enough levels of vitamin D_3_ appeared to be suggesting a reasonable mechanism for protection from MS by dietary modulations. Thus far, the roles of IL-27 and TGF- β1 and their association with other cytokines in MS disorder as a heterogeneous and complex autoimmune have been inconsistent and contradictory. Moreover, surveys are implicated in confirming the present model and illuminating the precise effect of IL-27 and TGF- β1 in terms of amelioration or development of MS. More probably, epigenetic nutritional mechanisms may be a milestone in finding a solution to such contradictions. Further, recent studies have also suggested that interactions between environmental factors, including vitamin D_3_, bioactive components, and hormones with epigenetic parameters are of principal reasons for MS. Because of their beneficial impressions on crucial TF engaged in neurodegeneration and axonal damage, dietary factors are also remarkably modifiable. Therefore, further investigations with using complementary and novel techniques such as flow cytometry, as well as taking into consideration of correlation the observed changes in cytokines to clinical and psychological measures of disease (EDSS, DASS, MRI measurements, etc.) alongside real time-PCR and ELISA are essential to improve therapeutic approaches and deal with all contradictions regarding the molecular mechanisms preoccupied in MS [49, 50].

The most obvious finding to emerge from this study was that the up-regulation of IL-27 and TGF- β1 could occur at adequate serum levels of vitamin D_3_, exclusively in MS patients. As stated, IL-27 and TGF- β1 could have positive feedback on the up-regulation of IL-10, and conversely, they could also have negative feedback on the up-regulation of IL-17A and IL-6. Due to this reason, IL-27 and TGF-β1 could have a major role in the immunomodulatory function and also could contribute to the prevention of the pathophysiological process of MS.

## Supporting information

S1 Table Interleukin ΔCT and vitamin D3 serum levels (ng/ml) at baseline and after eight weeks of supplementation. (DOCX)

S1 Data (ZIP)
