# Supplementary material for: The impact of vitamin D3 intake on inflammatory markers in multiple sclerosis patients and their first-degree relatives
Source: PLoS One. 2020 Apr 6;15(4):e0231145. doi: 10.1371/journal.pone.0231145 (PMC7135246; doi:10.1371/journal.pone.0231145)
Supplement: S1 Table — (DOCX) [file pone.0231145.s001.docx]

| S1 Table. Interleukin ΔCT and vitamin D3 serum levels (ng/ml) at baseline and after eight weeks of supplementation | | | | | | | | | |
| --- | --- | --- | --- | --- | --- | --- | --- | --- | --- |
| Variables | **MSP, n=25** | | **FDRP, n=25** | | **HP, n=25** | | **P^*^** | | |
|  | **Before** | **After** | **Before** | **After** | **Before** | **After** | **Before** | **After** | |
| vitamin D_3_ (mean ± SD)  P^**^  P^***^ | 25.94±9.49,  AB=0.067  - | 62.24±26.07,  AB=0.022  <0.001 | 18.46±12.09,  BC=0.890  - | 46.33±18.72,  BC=0.887  <0.001 | 19.98±13.07,  AC=0.174  - | 43.60±15.86,  AC=0.006  <0.001 | 0.063 | | 0.004 |
| IL-17A (mean ± SD)  P^**^  Fold changes†  P^***^ | 5.13±1.32,  AB=0.009  -  - | 6.16±1.93,  AB=0.022  -5.9  0.014 | 6.24±1.28  BC=<0.001  -  - | 7.43±1.51,  BC=0.001  -5.2  0.006 | 8.16±1.26  AC=<0.001  -  - | 9.23±1.44,  AC=<0.001  -4.2  0.012 | <0.001 | | <0.001 |
| IL-10 (mean ± SD)  P^**^  Fold changes  P^***^ | 7.93±1.66,  AB=<0.001  -  - | 5.97±2.52,  AB=0.016  6.2  0.005 | 9.82±1.38,  BC=0.025  -  - | 7.55±1.70,  BC=0.067  4.6  <0.001 | 10.87±1.07,  AC=<0.001  -  - | 8.82±1.56,  AC=<0.001  5.2  <0.001 | <0.001 | | <0.001 |
| IL-6 (mean ± SD)  P^**^  Fold changes  P^***^ | 4.73±1.09,  AB=<0.001  -  - | 5.99±2.00,  AB=<0.001  -5.6  0.003 | 6.79±1.38,  BC=<0.001  -  - | 8.15±1.17,  BC=<0.001  -5.5  0.002 | 8.74±1.04,  AC=<0.001  -  - | 10.21±1.40,  AC=<0.001  -5.1  <0.001 | <0.001 | | <0.001 |
| Data are presented as mean ± SD. Between-group comparisons were assessed by using one-way ANOVA, followed by post hoc Tukey's test. Within-group comparisons of IL-17A, IL-10, and IL-6 levels were performed by using paired t-test. P*, P**, and P*** indicate differences between-groups, pairwise comparisons (Tukey), and within-group differences, respectively. MSPs, FDRPs, and HPs were represented by A, B, and C, respectively. P<0.05 indicates statistical significance. The sign † indicates the expression levels of ILs, compared to the baseline values, based on ratio formula (2-ΔΔCT). Abbreviations: ANOVA, analysis of variance; IL, Interleukin; MSP, Multiple Sclerosis Participants; FDRP, First-Degree Relative Participants; HP, Healthy Participants. | | | | | | | | | |
